# Nearl: extracting dynamic features from molecular dynamics trajectories for machine learning tasks

**DOI:** 10.1093/bioinformatics/btaf321

**Published:** 2025-05-29

**Authors:** Yang Zhang, Andreas Vitalis

**Affiliations:** Department of Biochemistry, University of Zurich, Zurich, 8057, Switzerland; Department of Biochemistry, University of Zurich, Zurich, 8057, Switzerland

## Abstract

**Summary:**

Despite the rapid growth of machine learning in biomolecular applications, information about protein dynamics is underutilized. Here, we introduce Nearl, an automated pipeline designed to extract dynamic features from large ensembles of molecular dynamics trajectories. Nearl aims to identify intrinsic patterns of molecular motion and to provide informative features for predictive modeling tasks. We implement two classes of dynamic features, termed marching observers and property-density flow, to capture local atomic motions while maintaining a view of the global configuration. Complemented by standard voxelization techniques, Nearl transforms substructures of proteins into three-dimensional (3D) grids, suitable for contemporary 3D convolutional neural networks (3D-CNNs). The pipeline leverages graphics processing unit (GPU) acceleration, adheres to the FAIR principles for research software, and prioritizes flexibility and user-friendliness, allowing customization of input formats and feature extraction.

**Availability and implementation:**

The source code of Nearl is hosted at https://github.com/miemiemmmm/Nearl and archived at https://doi.org/10.5281/zenodo.15320286. The documentation is hosted on ReadTheDocs at https://nearl.readthedocs.io/en/latest/. All pre-built models are implemented in PyTorch and available on GitHub.

## 1 Introduction

Recent advances in machine learning (ML) have significantly reshaped the landscape of protein structure prediction (Baek *et al.* 2021, Jumper *et al.* 2021, Zheng *et al.* 2021), protein design (Dauparas *et al.* 2022), and generative drug discovery (De Cao and Kipf 2018, Gómez-Bombarelli *et al.* 2018), and expand traditional computational biology methods by incorporating techniques ranging from shallow learning to complex neural networks and even reinforcement learning. Three-dimensional (3D) voxels represent the spatial distribution of atoms within a molecule, essentially functioning as 3D images (Maturana and Scherer 2015). 3D convolutional neural networks (3D-CNNs) apply the convolution operation to 3D space and offer a popular framework for tasks such as predicting protein-ligand binding affinities (Jiménez *et al.* 2018, Stepniewska-Dziubinska *et al.* 2018, Hassan-Harrirou *et al.* 2020, Townshend *et al.* 2020, McNutt *et al.* 2021), protein-protein interfaces (Townshend *et al.* 2019, Crocioni *et al.* 2024), or binding pockets (Simonovsky and Meyers 2020).

Molecular dynamics (MD) simulations are an established and widely used tool to predict the evolution of a molecular system based on physical laws (McCammon *et al.* 1977, Karplus and McCammon 2002). They allow atomic-level exploration of the conformational space of biomolecules, offering potential insights and guidance for the design of ligands and biomolecules, the interpretation of experimental results, or understanding the dynamic behavior of proteins (Hollingsworth and Dror 2018). Several strategies have been developed to bridge the gap between the information contained in MD trajectories and the requirements posed toward inputs of machine learning frameworks. The high dimensionality of the phase space sampled in MD is addressed, in part, through dimensionality reduction and information condensation techniques. This is often achieved by calculating a set of simple collective variables (CVs) that effectively capture the essential dynamics of the system (Riniker 2017, Cocina *et al.* 2020, Morishita 2021, Gorostiola González *et al.* 2023). These techniques are frequently interfaced with established approaches toward time series modeling, which, unlike CNNs, rest on strong physical assumptions (Husic and Pande 2018, Grogan *et al.* 2020, Lickert and Stock 2020). In recent years, alternative methods for 3D feature embedding have been proposed, which move beyond traditional CVs, such as geometric bit vectors (Kumar *et al.* 2024), atom-centered symmetry functions (Gallegos *et al.* 2024), Coulomb matrices (Farahvash *et al.* 2020), and 2D grayscale images of radial distribution functions (Ayush *et al.* 2023).

Due to the data-hungry nature of deep learning frameworks (Adadi 2021), MD trajectories present a putatively valuable resource, both for data augmentation of the experimentally visible, conformational space and for incorporating information about dynamics. However, mining patterns of collective motion from MD trajectories is demanding due to their size (the biopolymers alone routinely contain up to tens of thousands of atoms, and individual trajectories contain similar numbers of frames) and heterogeneity across components (polymers vs solvent vs small molecules). Thus, using MD data to derive 3D features requires significant investments in computational power, file input/output operations, and data storage. The recent emergence of large-scale MD trajectory datasets (Meyer *et al.* 2010, Rodríguez-Espigares *et al.* 2020, Beltrán *et al.* 2024, Siebenmorgen *et al.* 2024, Vander Meersche *et al.* 2024) has significantly improved the accessibility of MD results for researchers. Consequently, there is a need for a framework that facilitates batch processing of MD trajectories to extract features that are explicitly aware of the underlying dynamics. The majority of 3D-CNN frameworks require specialized voxelization schemes (Stepniewska-Dziubinska *et al.* 2018, Hassan-Harrirou *et al.* 2020) that are not universally transferable to other applications. While tools like DeepRank (Crocioni *et al.* 2024) and libmolgrid (Sunseri and Koes 2020) systematically address the voxelization of protein-protein interactions and atomic properties, they mainly focus on static structures and do not explicitly respect the dynamic behavior of proteins in time. In this work, we present Nearl, an open-source Python package designed to streamline the extraction of both dynamic and static features from extensive MD trajectory datasets. The ultimate goal of Nearl is to provide a customizable and scalable solution for handling dynamic processes in 3D biomolecules, thereby achieving closer integration between MD and ML. We posit that MD data are underutilized, and Nearl aims to enhance the reusability of these data, in particular by state-of-the-art data-driven methods.

## 2 Featurization workflow

**Figure 1.**
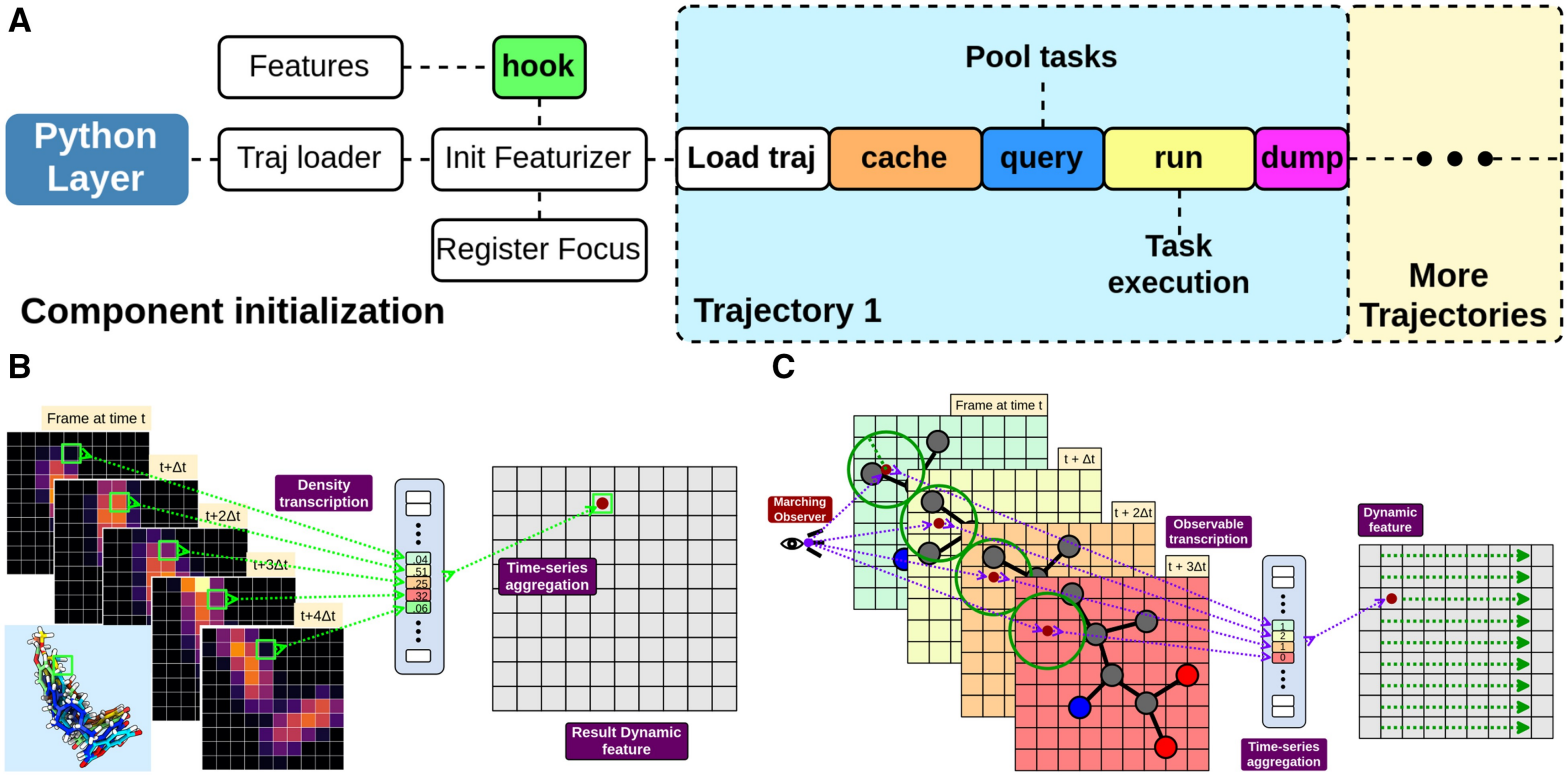
The architecture of featurization in Nearl and schematic 2D representation of the property density flow and marching observer algorithms processing sequential frames from a trajectory. (A) The overall workflow for the featurization pipeline for multiple trajectories. From left to right, the “Traj loader” registers the scope and type of structural input data to expect. This knowledge is needed to allow dynamic loading of the data by the “Featurizer.” As additional inputs, it requires user-requested “Features,” connected via “Hook,” as well as a definition of “Focus,” normally a geometric definition of a binding site. With these required inputs in place, the “Featurizer” can load different trajectories in chunks and cache the chunks. The remaining three child methods are “query,” “run,” and “dump.” The role of querying is to reduce a loaded chunk to isolate the region of interest. The queried data are pooled before the actual feature calculation can proceed (“run”). The calculated features are then made available, for example, written to a file (“dump”). (B) Property-density flow. Each frame is first voxelized onto a user-defined grid (10×10 in this example), and each grid point forms a time series of a rasterized property. An aggregation function is applied to summarize the dynamic change of the property over time. The 2D images are slices of a specific X−Y plane in a trajectory for convenience of demonstration (the full grid is cubic), and a superposition of 3D visualizations of the molecule across the time axis is shown in the lower left corner. (C) Marching observer. 2D illustration of the marching observer algorithm. The observer is marked by a dot in the center of a grid cell and records the presence of atoms (see the ball-and-stick representation) in its receptive field (the surrounding circle). The arrows allude to the fact that the same computations are done for every center point, meaning every grid point is an observer. The series of four frames represents consecutive snapshots of atomic coordinates.

Nearl adopts a modular and flexible approach for 3D feature generation. Four hierarchical layers are implemented in the feature extraction workflow from high to low levels: trajectories, frame-slices (a series of structural ensembles that share a common topology and cover a specific time span), substructures of interest, and features. The featurizer is the core module that coordinates other components and controls the trajectory loading, parses substructures of interest, and distributes frame slices to desired features. Feature level polymorphism utilizes five named child methods (hook, cache, query, run, dump) to customize the featurization process (see Fig. 1A). In this order, they are used to: (i) define global perception settings (subsystem size, etc.); (ii) cache properties (one-time); (iii) query the subsystem from frame-slices; (iv) compute features; (v) dump results. An arbitrary number of desired features can be registered for customizing the feature pool. The trajectory loader dynamically loads trajectories, splits them into frame slices (each slice is one task), and supplies them to the subsystem query (“query”). These tasks are pooled to enhance computational efficiency and reduce potential overhead in trajectory processing. The supported atomic features are listed as Table S1, available as supplementary data at *Bioinformatics* online. The “run” method then calculates various forms of results and potentially offloads computing-intensive features to GPU kernels. Depending on the nature of the output, we currently support static features (one frame —> one feature), dynamic features (frame-slice —> one feature), and labeling.

## 3 Features extraction from raw trajectories

### 3.1 Static features

The 3D voxel is a commonly used molecular representation that explicitly encodes molecular geometry and is suitable for 3D-CNNs. By mapping from Euclidean space to a discrete space, the substructure of interest, such as a ligand binding site or a molecular interface enclosed by a bounding box, is characterized. Equation (1) defines the voxelization based on distance-based Gaussian interpolation, similar to the technique employed in DeepRank (Renaud *et al.* 2021):


(1) $$w_{i,j,k}=\frac{1}{{\left(\sigma \sqrt{2\pi }\right)}^{3}}\sum_{a=1}^{N}w_{a}\;\text{exp}(-\frac{{\left\|c_{i,j,k}-p_{a}\right\|}^{2}}{2\sigma^{2}})$$

where *N* denotes the number of atoms, $w_{a}$ is the property or weight of atom *a*, $c_{i,j,k}$ represents the Cartesian coordinates of the grid point with internal index *[i*, *j*, *k]*, $w_{i,j,k}$ is the computed property density at this grid point, and σ is the standard deviation of the Gaussian kernel. Employing different types of weights (*w*) can populate different feature channels for the 3D-CNN framework for static structures. To capture the heterogeneity of molecules, 18 built-in, atomic properties are supported, such as atom type, topological flags, or partial charge (see Table S1, available as supplementary data at *Bioinformatics* online). OpenBabel (O'Boyle *et al.* 2011), RDKit (https://www.rdkit.org), and/or ACC II (Raček *et al.* 2020) are used to process intermediate substructures and compute required chemical properties. It is necessary to be aware of the level of approximations and ambiguities in these computations, *e.g.,* for the assignment of aromaticity or hydrophobicity. Since atoms are enumerated exhaustively and have, in principle, infinite support, the discretization requires a cutoff to save computational cost. Importantly, the use of a bounding box can lead to edge artifacts. To circumvent systematic edge truncation, Nearl buffers the bounding box by a number of voxels in all dimensions, covering the chosen cutoff. This means that a significantly larger subvolume is analyzed, but the computed features are only for the central part that is free of edge artifacts.

### 3.2 Dynamic features

The extension of the voxelization of static structures to a series of (consecutive) MD frames yields a series of 3D lattices. For each grid point with internal coordinates *i*, *j*, and *k*, a time series of discretized properties $\left\{v_{i,j,k}=w_{t,i,j,k}|t\in 1\cdots W\right\}$ is generated, and the dynamic property $v_{i,j,k}$ is computed by applying an aggregation function across nonoverlapping segments of length *W* to this time series (see Fig. 1B). With a choice of window size (*W* in the equation above), this gives rise to T/W aggregated observations where *T* is the total length of the trajectory in question. Supported aggregation functions are listed in Table S2, available as supplementary data at *Bioinformatics* online. We call this method “property-density flow,” PDF, and its elementary operation, voxelization of a single frame, is, naturally, implemented as in Section 3.1. The most expensive grid-based computations, such as Gaussian interpolation and time-series aggregation, can be accelerated by Compute Unified Device Architecture (CUDA) on NVIDIA GPUs, although it must be kept in mind that the overhead is not always justifiable, in particular for simple features and narrow frame slices.

We provide a second class of dynamic features we term “marching observer,” OBS, inspired by the marching cubes algorithm (Lorensen and Cline 1987). Here, every grid point serves as an observer making frame-by-frame “observations” of its immediate environment, the so-called receptive field. It monitors which atoms are found in its field to capture the local, instantaneous dynamics (shown in Fig. 1C). The observation functions are free choices and can be tailored to the specific research task. The observations are classified into direct counts of particles (presence of particles, number of atoms, etc.), or summary statistics of spatial configurations of particles (radius of gyration, mean distance to the observer, etc.). Table S3, available as supplementary data at *Bioinformatics* online lists the observable types to quantify the local configuration of particles in the receptive field. Each observer is subject to a cutoff distance that limits the perception of particles to this receptive field, and the corresponding observable is computed over the positions and weights of atoms without any special adjustments for molecular topology. This logic leads to discrete jumps when a particle crosses the cutoff distance. Taken together, the set of observations made by all observers continues to encode the overall configuration of the substructure of interest, but in a way that is less stringent and restrictive than for property-density flows. Note that the buffering of the grid happens here as well, using the receptive field’s radius to set the buffer size. The detailed algorithms for the two dynamics features (Algorithm S1 and S2) are available as supplementary data at *Bioinformatics* online.

Importantly, any given choice of feature and aggregator carries limited dynamical information, and the nature of this information will depend astutely on the size of the time window. While simple aggregation techniques have been shown to make a dramatic impact in weighting features (Blöchliger *et al.* 2015), it is also true that even in physics-based techniques, such as Markov state models, the processing of high-dimensional data from MD remains a formidable task offering few guarantees (Bacci *et al.* 2019, Kozlowski and Grubmüller 2023, Widmer *et al.* 2023). It is therefore not surprising that deep ML approaches are deployed merely as parts of a pipeline (Mardt *et al.* 2018) or that time-sensitive (recurrent) network architectures are evaluated for explicit dynamic comprehension (Tsai *et al.* 2020). Instead, all of Nearl’s featurization methods convert user-defined substructures into 3D grid representations directly suitable for 3D-CNN frameworks. As such, it is important to consider the physical and mathematical meanings of different combinations. Observations such as the presence or direct counts of particles are suitable for index-based properties, including residue indices, atomic indices, and annotations of different subparts of a system (*e.g.*, ligand *vs* receptor). For example, using the combination of ligand annotation and particle count, and using the mean to aggregate, we obtain the average count of ligand particles in the vicinity of a specific observer. It is then up to the choice of window size, *W*, to determine the time scale of fluctuations captured by this feature. The same will hold, albeit less obviously, for other cases. Consider, for example, a weight-like property such as atomic mass monitored by an observer in terms of distance to the observer. If we use the standard deviation to aggregate, we obtain a measure of variability for the mass-weighted distance of particles to the observer. Just as before, this measure of variability is time-local as prescribed by *W*.

As shown in Fig. S1, available as supplementary data at *Bioinformatics* online, we used the ligand binding constant ($pK_{i}$ or $pK_{d}$) prediction to offer a preliminary exploration of the effect of dynamic features. The experiment utilized the PDBBind refined (Wang *et al.* 2004) set as the training set, CASF2016 (Su *et al.* 2019) as the test set, and the trajectories are from the MISATO dataset (Siebenmorgen *et al.* 2024). Combining static with dynamic features tends to very slightly improve the models’ ranking capability, an observation found similarly in Table 3 in a recent work (Guo *et al.* 2025). On the flip side, using dynamic features alone leads to models that perform worse than static features alone. In this particular case, all dynamic features were aggregated using the sample standard deviation, and this removes, to an extent, the representation of the distribution itself. Gauging the impact of a feature *a priori* appears out of reach, and an extensive hyperparameter exploration will usually be needed. We reemphasize that Fig. S1, available as supplementary data at *Bioinformatics* online, is merely an illustration, and we currently do not feel equipped to make further-reaching statements about the potential impact of dynamic information in ML tasks.

### 3.3 Benchmark of feature generation and query

Due to its modular design, Nearl is currently not optimized for specific problems or problem sizes. However, the main cost-controlling factors, total data size, number of atoms in the focus region, and number of grid points, all impact the scaling. Due to the overhead of launching CUDA kernels, the exact mode of operation has a big impact on net throughput, which extends to aspects like the choice of (raw) input format, output settings, etc. Although both libmolgrid and DeepRank utilize similar grid-based frameworks, they rely on a predefined set of feature encodings and their own data-loading pipelines. In contrast, Nearl employs a fully modular approach: every feature, even one-hot encoding, is computed independently. This modularity would make a direct comparison between Nearl and these frameworks challenging to interpret.

From the data in Table 1, it is clear that the cost scales almost cubically with the number of grid points in one dimension. This is because the loops are written to allow parallelization in voxel (rather than atom) space, which is inefficient for large grids. For small grids, the constant cost of memory and GPU management is evident. The implementation of OBS is generally faster than that of PDF. Overall, a minimal cost per frame in the mid-μs range is achievable also with CPU-only processing (not shown), and future work is needed to make full use of CUDA within Nearl. A particular focus would be to find ways to reuse allocated memory, also on the GPU, without breaking Nearl’s modularity and inner logic. That said, the reported costs are below the typical cost of file-based I/O for biomolecular systems, which means that the practical ceiling might be quite limited.

**Table 1.** Benchmark of the generation of features at different grid resolutions (“run” module in Fig. 1).^a^

Grid size	Static	OBS	PDF
Grid 16^3^	0.153 (0.066)	0.033 (0.002)	0.033 (0.01)
Grid 24^3^	0.33 (0.224)	0.038 (0.007)	0.061 (0.033)
Grid 32^3^	0.671 (0.531)	0.045 (0.016)	0.113 (0.078)
Grid 48^3^	1.985 (1.793)	0.077 (0.055)	0.313 (0.265)
Grid 64^3^	4.466 (4.249)	0.16 (0.131)	0.695 (0.628)
Grid 96^3^	14.619 (14.34)	0.466 (0.441)	2.181 (2.118)
Grid 128^3^	33.992 (33.992)	1.044 (1.044)	5.02 (5.02)

a The cost per frame in *ms* is reported, with the number in parentheses the prediction from the largest grid ($128^{3}$) based on cubic scaling with the number of grid points in one dimension. Each frame contains synthetic data for 300 dummy atoms, and each frame-slice holds 50 frames (relevant only for dynamic features). The grid resolution was set to 1 Å, and the cutoff was fixed to 2.5 Å. No buffering was performed so that the nominal grid size in the table is the actual one. The evaluation for every grid size was performed at least 50 times.

## 4 Feature extraction, storage, and supply

The “trajectory loader” module utilizes PyTraj as the backend (https://github.com/Amber-MD/pytraj) for dynamically loading trajectories. Hence, it accommodates commonly used trajectory file formats such as NetCDF (Amber), XTC (GROMACS), and DCD (CHARMM, NAMD). The “trajectory” module can be customized to support deviating formats and standards by implementing child classes to load them. Formally, Nearl regards a static structure as a trajectory containing only one frame slice with a single frame. An arbitrary number of trajectories can be registered for feature extraction. However, due to the innate heterogeneity of MD data, *e.g.*, in atom names, atom order, or boundary conditions, the trajectories have to be preprocessed to arrive at a consistent schema, and we use CAMPARI for some of these tasks (https://campari.sourceforge.net/V5). It is particularly important that moieties of interest are annotated consistently and, for dynamic features, that the time intervals are resampled to be as homogeneous as possible. These points, along with the general compatibility of different trajectories in terms of MD settings (*e.g.*, temperature), are properties of the data and largely out of scope for Nearl. The resultant features are stored in Hierarchical Data Format (HDF) due to its high-performance access via h5py (https://github.com/h5py/h5py). Nearl employs process pools for data retrieval and allows concurrent access to multiple HDF data files (if they follow a consistent dataset schema). Users can easily switch to alternative storage media by modifying the “dump” method in a customized “feature” module.

While it is common practice to engineer features for specific biological tasks, their rigorous validation is frequently lacking, and systematic comparisons across diverse ML architectures and featurizations are seldom attempted. Furthermore, customization can be difficult to integrate into standardized, external ML workflows. In Nearl, the modularized and polymorphic design allows users to easily construct their feature engineering pipelines and perform validation across different workflows. Both training data and labels are stored as orthogonal datasets in HDF files. During training, multiple dataset tags can be queried for setting the training data. For seamless integration with deep learning frameworks, several pre-built 3D-CNNs are implemented within the PyTorch (Paszke *et al.* 2019) framework. These models are adapted from established architectures (Stepniewska-Dziubinska *et al.* 2018, Townshend *et al.* 2020, McNutt *et al.* 2021, Crocioni *et al.* 2024).

## 5 Conclusions

Nearl aims to narrow the gap between deep learning applications and MD simulations. We focus here on CNNs as algorithms for explicit 3D spatial perception tasks rather than on graph-based neural networks (GNNs). Of course, “spatial topology” can also be encoded as a graph by using larger thresholds for defining “bonds,” and we expect that such GNNs will offer similar performance to CNNs, see for example (Guo *et al.* 2025). We believe that Nearl contributes to enhancing the reusability of MD trajectories, thereby unlocking their full potential for informing and accelerating biological research. Nearl’s design prioritizes user customization and extensibility, which allows researchers to seamlessly integrate their own features and models. As the public availability of curated MD data is steadily increasing, we anticipate that the software will be a valuable tool for computational biologists, bioinformaticians, and machine learning engineers. By providing full support for “4D” data from MD (dimensions of *X*, *Y*, *Z*, and time), it offers an alternative paradigm to most other ML approaches designed for 3D structures (Sunseri and Koes 2020, Crocioni *et al.* 2024). Researchers can deploy Nearl to incorporate information about biomolecular dynamics into diverse areas of investigation, including drug discovery, molecular design, and structural biology.

While the theoretical usefulness of data reporting on dynamic aspects of biological systems is indisputable, it is clear that this potential is not straightforward to unlock in practice (compare Fig. S1, available as supplementary data at *Bioinformatics* online). In particular, it might be harder for tasks such as affinity prediction than for, *e.g.*, binding site identification (Guo *et al.* 2025). It is fair to expect limited returns if the relation between fluctuations and their rates and the target property is complex. For affinity prediction, on average, larger deviations in pose occur for weaker binders on short time scales, as expected (Marchand *et al.* 2020), but this is clearly of limited predictive value since buriedness, ligand size, and ligand flexibility will all codetermine the scale of observed fluctuations, both spatially and temporally. A further caveat is the tendency of deep ML models to learn non-transferable means of solving the task (Zhang and Vitalis 2025). Given the sparsity of data in areas such as binding affinity, models would have to overcome this overfitting, which in essence corresponds to finding a specific set of rules for every training data point, for the inclusion of dynamical information to deliver the expected impact.

## Supplementary Material

btaf321_Supplementary_Data

## Data Availability

The source code repositories underlying this study are available at https://github.com/miemiemmmm/Nearl and https://doi.org/10.5281/zenodo.15320286.
