# Supplementary material for: Nearl: extracting dynamic features from molecular dynamics trajectories for machine learning tasks
Source: Bioinformatics. 2025 May 29;41(7):btaf321. doi: 10.1093/bioinformatics/btaf321 (PMC12233089; doi:10.1093/bioinformatics/btaf321)
Supplement: btaf321_Supplementary_Data [file btaf321_supplementary_data.zip › Nearl_SI_proof_correction.pdf]

Supplemental Information

Built-in atomic properties

Table S1: **Built-in property types in Nearl**

Property type	Data type	Source
Atomic ID	Integer	Topology
Residue ID	Integer	Topology
Atomic number	Integer	Topology
Hybridization	Integer	OpenBabel
Selection	Integer	Topology and user input
Mass	Float	Periodic table
Radius	Float	Periodic table
Electronegativity	Float	Periodic table
Hydrophobicity	Float	Predefined function
Partial charge	Float	Topology or external software
Heavy atom	Boolean	Topology
Aromaticity	Boolean	OpenBabel
Ring	Boolean	OpenBabel
Hydrogen bond donor	Boolean	OpenBabel
Hydrogen bond acceptor	Boolean	OpenBabel
Backbone	Boolean	Topology
Sidechain	Boolean	Topology
Atom type	One-hot	Topology

It is important to note that not all of these features are necessarily stable. For example, the assignment of aromaticity by OpenBabel depends on planarity, which is variable across a simulation trajectory. The use of such features should generally be reserved for specialized applications. Moreover, default values for hydrophobicity or electronegativity are primitive, element-based mappings, and users should replace them with their preferred versions.

Aggregation functions for dynamic features

Table S2: **Aggregation types available in Nearl**

Aggregation type	Weight type
Mean	Number-like
Standard deviation	Number-like
Median	Number-like
Variance	Number-like
Max	Number-like
Min	Number-like
Information entropy	Number-like or Indices
Drift	Number-like

Simple aggregation functions in Table S2, such as minimum, maximum, mean, variance, and (sample) standard deviation, are computationally straightforward. We implemented a histogram-based function to compute the **information entropy** of the observables. Firstly the minimum and maximum values in the observable array are found to determine the range of the data. Then, values are normalized to the range of 0 to 1 and binned into a histogram with B bins. The frequency of values in each bin is used to compute the probability distribution (p) of the observable array. The information entropy (H) is calculated using Equation S1.

$$H = -\sum_{i=0}^{B-1} p_i \log_2 p_i \tag{S1}$$

where p_i is the probability of the i-th bin and B is the hardcoded number (16) of bins. This entropy quantifies the uncertainty or randomness in the observable array, with a higher value indicating a more evenly distributed dataset, and a lower value indicating a more concentrated dataset.

For the **drift** aggregation function, the observables (y) and their indices (time-axis, x) are used to compute the drift using linear regression. The drift coefficient D is calculated as the slope of the linear regression line, as shown in Equation S2.

$$D = \frac{W\sum_{i=0}^{W-1} x_i y_i - \sum_{i=0}^{W-1} x_i \sum_{i=0}^{W-1} y_i}{W\sum_{i=0}^{W-1} x_i^2 - \left(\sum_{i=0}^{W-1} x_i\right)^2} \tag{S2}$$

where W is the window size (number of frames in the given frame slice), and x_i and y_i are the implicit time indices and observables at time i, respectively. The drift coefficient quantifies the linear trend in the time series, with positive values indicating increasing trends and negative values indicating decreasing trends.

Observable types for the marching observer algorithm

Table S3: **Observable types available in Nearl along with their data type and suitable weight types.**

Observation type	Observable datatype	Suitable weight type
Existence of particle	Boolean	N/A
Count of particles	Integer	N/A
Distinct count	Integer	Index-like
Mean distance	Float	Weight-like
Cumulative weight	Float	Weight-like
Density	Float	Weight-like
Eccentricity	Float	Weight-like
Radius of gyration	Float	Weight-like

The **existence of particles** quantifies whether or not at least one particle is found in the receptive field. This will typically be used in conjunction with a splitting of the data into separate channels for distinct entities, *e.g.*, protein and ligand. The **count of particles** instead counts the number of particles (n) in the receptive field. This will be closely related to the cumulative weight observer. The **distinct count** observer first splits the particles into possible classes, and these classes are usually determined from the unique values in a provided property, see Table S1. The observer than counts the number of unique classes with at least one particle in its receptive field.

Cumulative weight (W) represents the sum of all particles' weights within the receptive field of the observer, calculated as follows:

$$W = \sum_{i=1}^{n} w_i \tag{S3}$$

Density (ρ) is the cumulative weight normalized by the volume of the receptive field of each observer, with c being the radius (also cutoff) of the spherical receptive field. This ratio indicates the density of particles within the field, providing a measure of local particle concentration.

$$\rho = \frac{\sum_{i=1}^{n} w_i}{\frac{4}{3}\pi c^3} \tag{S4}$$

Mean distance (d) quantifies the average weighted distance between the observer p_0 and particle set p, providing insight into the spatial distribution of the particles relative to the observer. It is initialized to the cutoff value defining the observer's receptive field (rather than to 0.0).

$$d = \frac{\sum_{i=1}^{n} w_i \left\| p_i - p_0 \right\|}{\sum_{i=1}^{n} w_i} \tag{S5}$$

Eccentricity (E) measures the alignment of the particle set with respect to the observer, calculated as the distance between the center of mass (COM) of the particles and the observer's position.

$$E = \left\| COM(p) - p_0 \right\| \tag{S6}$$

Radius of gyration (ROG) provides a measure of the distribution of particles around its center of mass.

$$ROG = \sqrt{\frac{\sum_{i=1}^{n} w_i \left\| p_i - COM(p) \right\|^2}{\sum_{i=1}^{n} w_i}} \tag{S7}$$

Supplemental figures

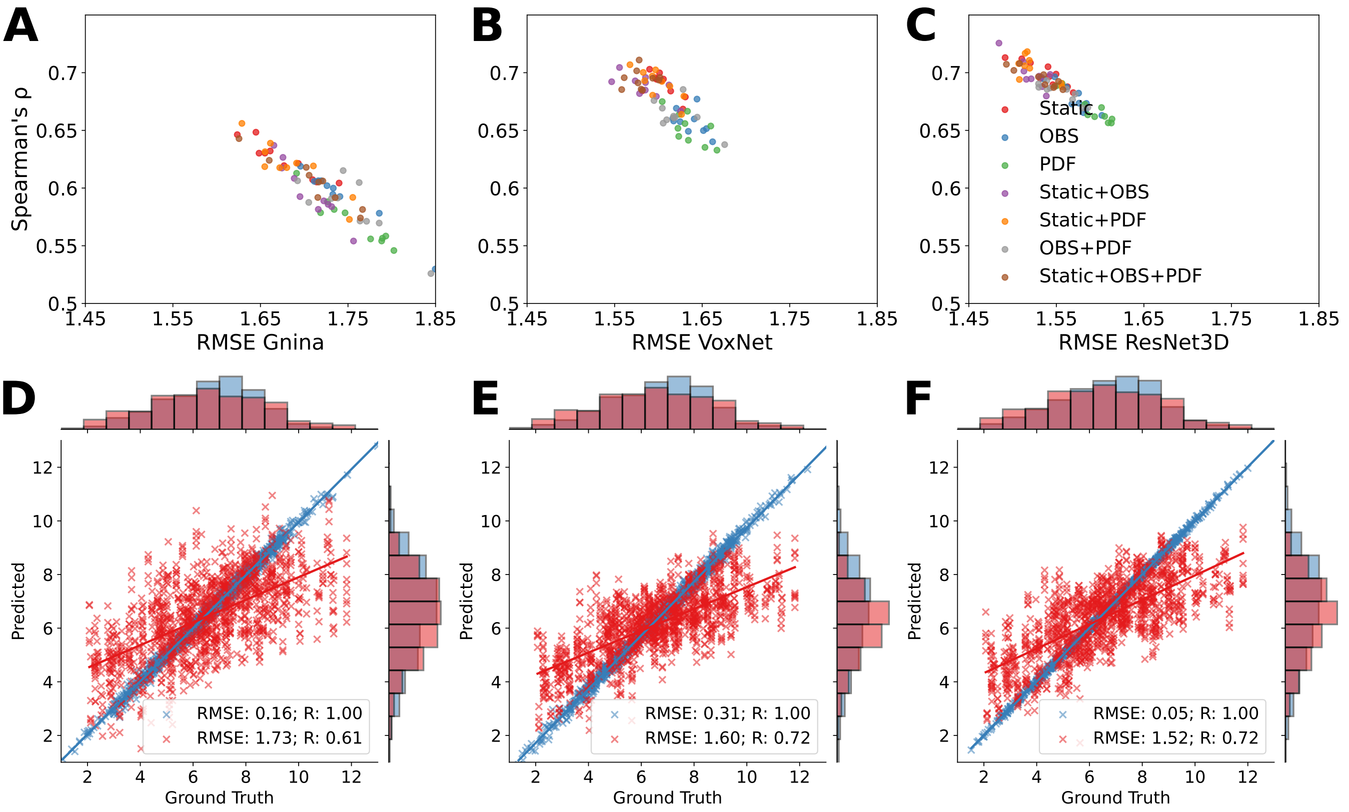

Figure S1: **Comparison of different combinations of static and/or dynamic features and evaluation of the prediction accuracy and generalizability on three tested models.** The tests are performed on the Misato dataset (see main text). The featurization used 32x32x32 grids with cubic voxels of 24 Å side length and five 20-frame windows per trajectory for OBS and PDF. For static features, the 10th frame in each window was selected. The cutoff (same as observer radius) and σ were 5.0 Å and 1.5 Å, respectively. The featurized property was mass but split into two channels: ligand and protein. Panels **A**, **B**, and **C** show scatter plots of the root mean square error (RMSE) against Spearman's ranking coefficient for 3 tested models: Gnina, VoxNet, and 3D ResNet. The legend in C applies to all three panels. Each feature combination was trained 10 times for 50 epochs each with little changes observed after 20 epochs. The results highlight that some differences across featurizations are systematic, albeit with considerable noise from the initialization. Generally speaking, there are two groups: dynamic features alone *vs* all sets including static features, with the latter performing systematically better for all architectures. The generalizability is poor throughout: the predictions against the ground truth for both training (blue dots) and test sets (red dots) are presented in panels **D**, **E**, and **F**. These models are the top-performing ones according to the last 10 epochs. All models overfit the training data to achieve near-perfect performance, which drops off dramatically for the test set. That said, Spearman correlations near 0.7 are excellent results for this type of test. OBS = marching observers; PDF = property-density flow.

Supplemental algorithms

Algorithm S1 Extract property-density flow feature in one frame-slice

Input: dims $d[3]$, coordinate set p, weight set w, frame number F, atom number A, spacing s, cutoff c, Gaussian standard deviation σ, aggregation function f_{agg}

Count (g') of grid points with buffering of its cutoff: g' = $(d_1 + 2(c+s)/s) \times (d_2 + 2(c+s)/s) \times (d_3 + 2(c+s)/s)$

Initialize: Temporary grid for 1 frame with buffering $R''[g']$

Count (g) of grid points: g = $d_1 \times d_2 \times d_3$

Initialize: Result grid $R[g]$

Initialize: Temporary grid for all frames $R'[g, F]$

for $f = 0$ **to** F **do**

  for $a = 0$ **to** A **do**

    Coordinate of atom a in the frame f: $r[3]$ = $p_{f,a}$

    for $i = 0$ **to** g' **do**

      $r'[3]$ = gridCoordinateWithBuffering(i, d, c, s)

      R''_i = gaussian($\|r' - r\|$, c, σ)

    end for

    Sum (s') the grid points for normalization: s' = $\sum R''$

    for $i = 0$ **to** g **do**

      $r'[3]$ = gridCoordinateWithoutBuffering(i, d, s)

      v_t = gaussian($\|r' - r\|$, c, σ)

      $R'_{i,f}$ = $(v_t \times w_{f,a})/s'$

    end for

  end for

end for

Initialize: Temporary time series $o'[F]$

for $i = 0$ **to** g **do**

  for $f = 0$ **to** F **do**

    $o'_f = R'_{i,f}$

  end for

  R_i = $f_{agg}(o')$

end for

Output: R

Algorithm S2 Extract marching observers feature in one frame-slice

Input: dims $d[3]$, coordinate set p, weight set w, frame number F, atom number A, spacing s, cutoff c, observation function f_{obs}, aggregation function f_{agg}

Count (g) of grid points (observers): g = $d_1 \times d_2 \times d_3$

Initialize: Result grid $R[g]$

Initialize: Temporary grid for all frames $R'[g, F]$

for $f = 0$ **to** F **do**

  for $i = 0$ **to** g **do**

    $r[3]$ = gridCoordinate(i, d, s)

    Make observation on coordinates (p_f) in frame f: $R'_{i,f}$ = $f_{obs}(r, p_f, w_f, A, c)$

  end for

end for

Initialize: Temporary time series: $o'[F]$

for $i = 0$ **to** g **do**

  for $f = 0$ **to** F **do**

    $o'_f = R'_{i,f}$

  end for

  R_i = $f_{agg}(o')$

end for

Output: R
